# Taxonomic review of the genus *Tambinia* Stål (Hemiptera, Fulgoromorpha, Tropiduchidae) with descriptions of four new species from the Pacific region

**DOI:** 10.3897/zookeys.132.1571

**Published:** 2011-10-03

**Authors:** Rong-rong Wang, Ai-Ping Liang

**Affiliations:** 1 Key Laboratory of Zoological Systematics and Evolution, Institute of Zoology, Chinese Academy of Sciences, 1 Beichen West Road, Chaoyang District, Beijing 100101, China

**Keywords:** *Tambinia*, Tropiduchidae, Fulgoromorpha, new species, Pacific region

## Abstract

Four new species of *Tambinia* Stål (Hemiptera: Fulgoromorpha: Tropiduchidae), *Tambinia conus* **sp. n.** (Papua New Guinea), *Tambinia macula* **sp. n.** (Malaysia: Borneo), *Tambinia robustocarina* **sp. n.** (Malaysia: Sabah) and *Tambinia sexmaculata* **sp. n.** (Australia: Kuranda) are described and illustrated from the Pacific region. The diagnostic characters of this genus are redefined. A checklist and a key to the known species of *Tambinia* are provided.

## Introduction

The tropiduchid planthopper genus *Tambinia* was established by Stål (1859) for *Tambinia languida* Stål, *Tambinia debilis* Stål and *Tambinia rufoornata* Stål, all from Sri Lanka. The type species, *Tambinia languida* Stål, was fixed later by Distant (1906) by subsequent designation. *Tambinia* is currently placed in the tribe Tambiniini Kirkaldy, 1907 (Metcalf 1954; Fennah 1982). The tropiduchid tribe Tambiniini, as redefined by Fennah (1982), comprises ten genera, *Tambinia*, *Ossoides*, *Sumbana*, *Kallitaxila*, *Kallitambinia*, *Nesotaxila*, *Garumna*, *Paragarumna*, *Athestia* and *Biruga*. However, in a recent study about tribe Tambiniini, Wang et al. (2009) established one new genus *Garumnella*, and *Paragarumna* was placed as a junior synonym of *Garumna*. O’Brien (2010) also established one new genus *Diambon* in the study of New World Tambiniini from palms. Members of Tambiniini are mainly distributed in the tropical regions of the World.

Although maximum attention has been paid to the monophyly and phylogeny in Fulgoromorpha, relatively little is known about the monophyly of both the Tambiniini and *Tambinia* and their relationships with other tropiduchid taxa in a cladistic sense. Only few papers provided valuable information about *Tambinia*: Wilson (1986) has stated that the Oriental and Australasian genera *Nesotaxila* and *Kallitaxila* appear to be most closely related to *Tambinia*. Asche and Wilson (1989) have indicated that some similarity exists in the aedeagal structure in *Tambinia* species and *Ommatissus* Fieber, 1875 (Trypetimorphini). A cladistic analysis is needed, but is beyond the scope of this paper.

While sorting and identifying Tropiduchidae from material on loan from the California Academy of Sciences, San Francisco, California, USA (CAS), National Museum of Natural History, Smithsonian Institution, Washington, DC, USA (USNM) and elsewhere, we found four new species of *Tambinia* from Papua New Guinea, Malaysia (Borneo, Sabah) and Australia (Kuranda). A revised generic diagnosis and a checklist of all known species of *Tambinia* are provided. A key to known species is also updated.

## Materials and methods

Dry pinned specimens were used for the descriptions and illustrations. External morphology was observed under a stereoscopic microscope and characters were measured with an ocular micrometer. Abdomens were removed and macerated in cold 10% KOH overnight. Precise dissections and cleaning of genitalic structures were finished in distilled water. Observations and drawings were done in glycerine under a compound light microscope. Photographs of the types were taken with a Nikon Coolpix 5400 digital camera. The digital images were then imported into Adobe Photoshop 8.0 for labeling and plate composition. Line figures were drawn with the aid of a camera lucida mounted on a Zeiss Stemi SV-11 stereomicroscope.

Specimens of three previously described species of the genus *Tambinia*, i.e. *Tambinia bizonata* Matsumura, 1914, *Tambinia rubrolineata* Liang, 2003 and *Tambinia similis* Liang, 2003, have been examined. No specimens of the other seventeen previously described species were available for examination. However, there is no doubt concerning the identity of those species because the descriptions and illustrations were very clear and detailed. For detailed descriptions and figures of the seventeen previously described species, see (Distant (1906, 1916), (Fennah (1956, 1970, 1982), Ghauri (1976), Matsumura (1914), Melichar (1914), (Metcalf (1946, 1954), Men et al. (2009), Muir (1931), Wilson (1986) and Wilson and Malenovský (2007).

Specimens examined during the course of this study are deposited in the CAS, USNM and Bernice P. Bishop Museum, Honolulu, Hawaii, USA (BPBM). The terminology follows Bourgoin and Huang (1990) and Wang et al. (2009).

## Taxonomy

### Genus Tambinia

Stål, 1859

http://species-id.net/wiki/Tambinia

Tambinia Stål 1859: 316; Distant 1906: 276; Bierman 1910: 26; Muir 1931: 303; Metcalf 1954: 100; Liang and Jiang 2003. Type species *Tambinia languida* Stål by subsequent designation.
Ossa de Motschulsky, 1863: 106; Bierman 1910: 26.

#### Diagnostic characters.

**Figure 1.**
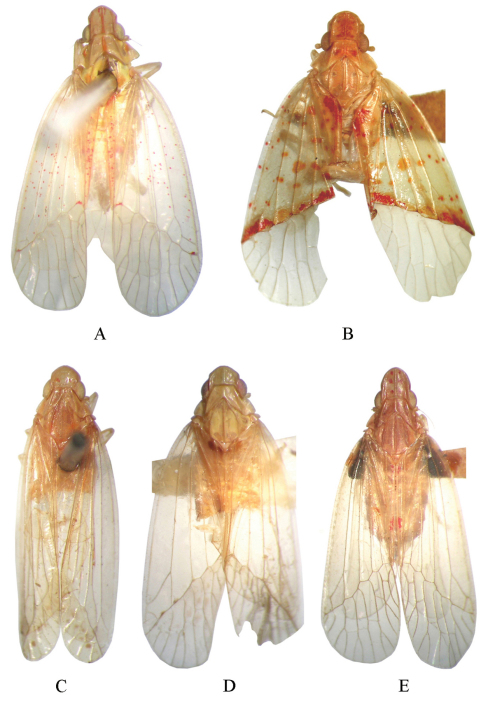
Dorsal habitus of *Tambinia* species **A** *Tambinia conus* sp. n. (male, Papua New Guinea, CAS) **B** *Tambinia macula* sp. n. (male, Malaysia:Borneo, CAS) **C** *Tambinia similis* Liang (male, Vietnam, BPBM) **D** *Tambinia robustocarina* sp. n.( male, Malaysia: Sabah, USUM) **E** *Tambinia sexmaculata* sp. n.( male, Australia:Kuranda, CAS).

**Figure 2.**
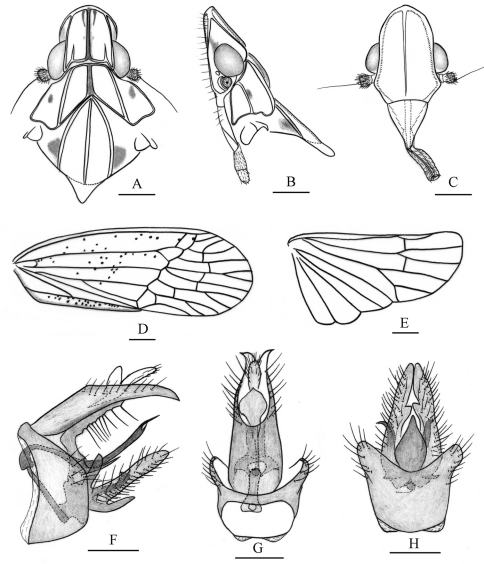
*Tambinia conus* sp. n. **A** head, pronotum and mesonotum, dorsal view **B** head, pronotum and mesonotum, lateral view **C** head, ventral view **D** right fore wing **E** right hind wing **F** male genitalia, left view **G** anal segment and pygofer, dorsal view **H** pygofer and gonostylus, ventral view. Scale bars: Figs A–C = 0.25 mm; D–E = 0.5 mm; F–H = 0.25 mm.

**Figure 3.**
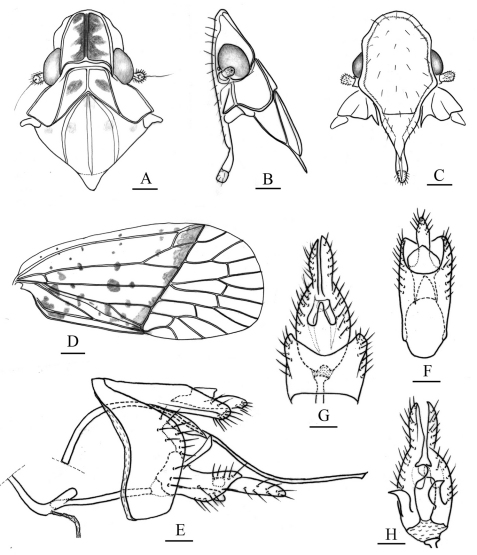
*Tambinia macula* sp. n.**A** head, pronotum and mesonotum, dorsal view **B** head, pronotum and mesonotum, lateral view **C** head, ventral view **D** right fore wing **E** male genitalia, left view **F** anal segment, dorsal view **G** pygofer and gonostylus, ventral view **H** gonostylus, dorsal view. Scale bars: Figs A–D = 0.25 mm; E–H = 0.125 mm.

**Figure 4.**
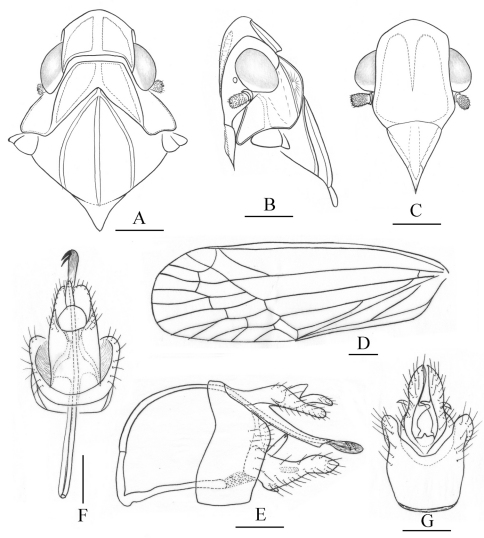
*Tambinia robustocarina* sp. n.**A** head, pronotum and mesonotum, dorsal view **B** head, pronotum and mesonotum, lateral view **C** head, ventral view **D** left fore wing **E** male genitalia, left view **F** male genitalia, dorsal view **G** pygofer and gonostylus, ventral view. Scale bars: Figs A–C = 0.25 mm; D = 0.5 mm; E–G = 0.25 mm.

**Figure 5.**
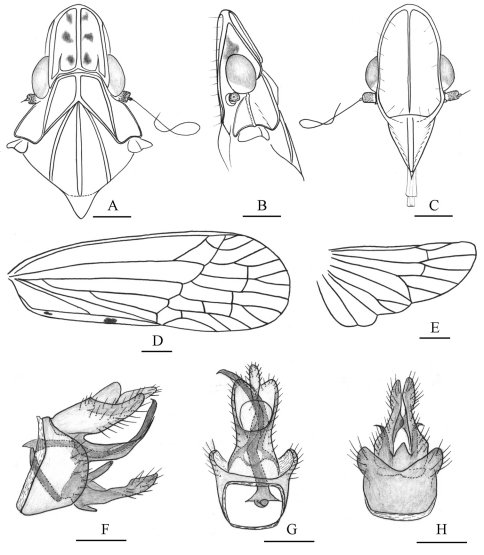
*Tambinia sexmaculata* sp. n. **A** head, pronotum and mesonotum, dorsal view **B** head, pronotum and mesonotum, lateral view **C** head, ventral view **D** right fore wing **E** right hind wing **F** male genitalia, left view **G** male genitalia, dorsal view **H** pygofer and gonostylus, ventral view. Scale bars: Figs A–C = 0.25 mm; D–E = 0.5 mm; F–H = 0.25 mm.

 Small-sized tropiduchids. Head (Figs 1A–E) with eyes narrowed than pronotum, distinctly produced in front of eyes and apically rounded, usually strongly dorsoventrally depressed and distinctly flattened in lateral view. Vertex (Figs 1A–E, 2–5A) tricarinate, disc of vertex (excluding median carina) depressed, posterior margin straight. Frons (Figs 2–5C) distinctly reclined caudad, somewhat flat and smooth, with or without median carina, rarely covered with sparsely microsetae. Clypeus triangular, relatively convex, with or without median carina, lateral margins not carinate. Rostrum short, not reaching mesotrochanters. Ocelli very small. Antennae with scape very small, pedicel cylindrical, covered with long setulae, sensory plaques present on top surface of pedical. Pronotum (Figs 2–5A) tricarinate, anterior margin straight and hind margin angulately excavate, with a single carina between eye and tegula. Mesonotum tricarinate. Hind titiae each with 2 distinct lateral spines; spinal formula of hind leg (4–5)–(4–5)–2; metatarsal segment II short and small. Forewings (Figs 1A–E) with oblique nodal line, apical portion flexing ventrad at this line, basal portion somewhat sub-hyaline, with or without granulate, thicker than apical portion, costal cell without cross veins.

Male genitalia. Pygofer (Figs 2F–H, 3E, 3G, 3H, 4E–G, 5F–H) symmetrical, dorsal margin deeply excavated to accommodate anal tube. Gonostylus (Figs 2F, 2H, 3E, 3G, 3H, 4E, 4G, 5F, 5H) elongate, bilaterally symmetrical, membranously fused with pygofer at base, with a conical, median process in ventral view, with a dorsally directed process arising from inner side near base and a laminate, inward directed, triangular process arising from inner side near middle. Periandrium (Figs 2F, 3E, 4E, 5F) dorsally connected with ventrobasal margin of anal tube, membranously fused with pygofer at ventral side, tube-like, distinctly sclerotized, surrounding aedeagus subapically or mesially, and visible in lateral view. Aedeagus (Figs 2F, 3E, 4E, 4F, 5F, 5G), asymmetrical, elongate and tubular, shaft of aedeagus (Figs 2F, 3E, 4E, 4F, 5F, 5G) slender and elongate, tubular, and sinuate in lateral view, subapically or mesially embraced in periandrium, endosoma membranous, with or without spines.

#### Discussion.

 The genus *Tambinia* comprises twenty-four species and is distributed in Oriental, Australasian and Afrotropical regions (Distant 1906, 1916, Fennah 1956, 1970, 1982, Ghauri 1976, Matsumura 1914, Melichar 1914, Metcalf 1946, 1954, Muir 1931, Wilson 1986, Wilson and Malenovský 2007). The tropiduchid planthoppers are usually weak fliers and have poor ability for long-distance migration by themselves. So, we indicate that new species have formed through geographical isolation over the disjunct distribution of the genus across widely separated island groups.

In external appearance, the genus *Tambinia* is similar to the Oriental and Australasian genera *Nesotaxila*, *Kallitaxila* and *Kallitambinia*. These four genera form a distinct group within tribe Tambiniini. They can be distinguished from the other known genera in the tribe by the head relatively dorsoventrally depressed, produced in front of eyes, but not extreme produced into a linguiform prolongation, apex not broadly rounded to base of frons, and hind tibia with two lateral spines. The four genera can be distinguished as follows:

**Table:** 

1	Two carinae on each side of pronotum between eye and tegula and an incomplete carina behind eye	*Nesotaxila*
–	At most only one complete and one incomplete carina on each side of pronotum between eye and tegula	2
2	One complete and one incomplete carina on either side of pronotum between eye and tegula; vertex with sublateral carinae distinct and stubby	*Kallitaxila*
–	A single carina laterally on pronotum between eye and tegula; vertex without sublateral carinae, if not, only slender sublateral carinae present	3
3	Forewings with corium granulate; anal tube extreme long, distinctly surpassing terminal of genitalia, aedeagus a simple tube with accompanying spike	*Kallitambinia*
–	Forewings with corium not granulate, or only obscurely granulation present; anal tube relatively short, not surpassing or slightly surpassing surpassing terminal of genitalia	*Tambinia*

##### Check list of species of Tambinia Stål

*Tambinia atrosignata* Distant, 1906; Sri Lanka (Paradeniya).

*Tambinia bizonata* Matsumura, 1914; China (Taiwan), Japan.

*Tambinia capitata* Distant, 1906; Burma, Malay States, India.

*Tambinia conus* sp. n.; Papua New Guinea.

*Tambinia debilis* Stål, 1859; India, Sri Lanka, Vietnam, South China (Anhui Province, Guangdong Province, Guangxi Zhuang Autonomous Region, Zhejiang Province, Fujian Province, Hainan Island, Hong Kong, Taiwan), Japan, Malacca, Malaysia, Singapore.

*Tambinia exoleta* Melichar, 1914; New Guinea (Moroka).

*Tambinia fasciculosa* Melichar, 1914; New Guinea (Moroka).

*Tambinia guamensis* Metcalf, 1946; Micronesia (Guam).

*Tambinia inconspicua* Distant, 1906; Burma.

*Tambinia languida* Stål, 1859; Sri Lanka.

*Tambinia macula* sp. n.; Malaysia (Borneo).

*Tambinia menglunensis* Men & Qin, 2009; China (Yunnan Province).

*Tambinia pitho* Fennah, 1970; Philippines.

*Tambinia robustocarina* sp. n.; Malaysia (Sabah).

*Tambinia rubrolineata* Liang, 2003; South China (Hainan Island), Laos, Vietnam.

*Tambinia rubromaculata* Distant, 1916; Sri Lanka.

*Tambinia rufoornata* Stål, 1859; Sri Lanka.

*Tambinia sexmaculata* sp. n.; Australia (Kuranda).

*Tambinia similis* Liang, 2003; Vietnam.

*Tambinia sisyphus* Fennah, 1956; Micronesia (Western Caroline Islands: Palau).

*Tambinia theivora* Fennah, 1982; Malaysia (Cameron Highlands).

*Tambinia venusta* (Kirkaldy, 1906); Australia (Queensland), New Guinea.

*Tambinia verticalis* Distant, 1916; India (Southern India, Coorg, Madras), Zanzibar, Tanga.

*Tambinia zonata* Muir, 1931; India (Madras).

##### Key to species of genus Tambinia

**Table:** 

1	Vertex shorter in middle than the widest breadth, or about as long as broad	2
–	Vertex distinctly longer in middle than the widest breadth	11
2	Frons with carina obsolete	3
–	Frons with carina distinct	4
3	Frons about as long as broad, forewings with two black elongate spots near bases of sutural margins, nodal line marked with several fuscous spots (see Distant, 1906: 278)	*Tambinia atrosignata* Distant
–	Frons (Fig. 3C) distinctly longer than broad, forewings (Figs 1B, 3D) with two red elongate marks near bases of sutural margins, many orange or red spots marked from basal part to nodal line, nodal line suffused with one transverse orange to red band	*Tambinia macula* sp. n.
4	Forewings with granulate	5
–	Forewings without granulate	6
5	Forewings marked without transverse bands (see Distant 1906: 279, Fig. 129; Distant 1906: 277)	*Tambinia debilis* Stål
–	Forewings marked with two brown transverse bands across wing sub-basally, on nodal line and in clavus (Yang et al. 1989: 80, Fig.6)	*Tambinia bizonata* Matsumura
6	Forewings with nodal line near apex	7
–	Forewings with nodal line near middle	9
7	Forewings with marks and stripes distinct	8
–	Forewings with marks and stripes very pale, nearly absent (Fig. 1C)	*Tambinia similis* Liang
8	Forewings with 11 apical cells, 4–5 subapical cells (see Liang, 2003: 511; Fig. 1)	*Tambinia rubrolineata* Liang
–	Forewings with 9 apical cells, 3–4 subapical cells (see Fennah 1982: 641, Fig. 35)	*Tambinia theivora* Fennah
9	Body suffused with distinct spots and markings	10
–	Body (Fig. 1D) without spots and markings, median carinae of vertex and pronotum thickened and broad, frons (Fig. 4C) with basal part of median carina strongly broad and thickened, not reaching to frontoclypeal suture, obsolete on level of antennae	*Tambinia robustocarina* sp. n.
10	Vertex, pronotum and mesonotum marked with reddish spots, forewings with nodal line suffused with red stripes (see Men and Qin 2009: 263, Figs 1, 2)	*Tambinia menglunensis* Men & Qin
–	Vertex without spots, pronotum with posterior margin marked with reddish stripes, mesonotum with carinae reddish, forewings with nodal line suffused with fuscous (see Distant 1906: 278)	*Tambinia rufoornata* Stål
11	Vertex medially 1.1–1.3 times as long as maximum breadth	12
–	Vertex medially 1.4–1.8 times as long as maximum breadth	18
12	Body above suffused with marks or different colors	13
–	Body above concolorous, without marks or different colors	16
13	Vertex with sublateral carinae basally between median carina and lateral margins	14
–	Vertex without sublateral carinae between median carina and lateral margins	15
14	Vertex (Figs 1E, 5A) with six red spots, pronotum and mesonotum without spots, forewings (Figs 1E, 5D) with two pairs of red spots near bases of sutural margins and distad of level of union of claval veins relatively	*Tambinia sexmaculata* sp. n.
–	Vertex (Figs 1A, 2A) with two short reddish stripes, pronotum with a pair of orange spots outside lateral carinae, carinae on vertex and pronotum orange, mesonotum with a pair of orange spots beside lateral carinae near posterior margin, forewings (Figs 1A, 2D) with many reddish spots marked from basal part to nodal line	*Tambinia conus* sp. n.
15	Carinae on vertex, pronotum and mesonotum without pigmentation, mesonotum suffused with ochraceous (see Distant, 1906: 276, Fig. 127)	*Tambinia languida* Stål
–	Carinae on vertex, pronotum and mesonotum reddish, mesonotum suffused with dark brown (see Muir, 1931: 303)	*Tambinia zonata* Muir
16	Head not prominently narrowed anteriorly	17
–	Head gradually narrowed to apex (see Distant 1906: 278)	*Tambinia capitata* Distant
17	Forewings with Cu_1_ forking distad of level of union of claval veins, with 12 apical cells, subapical cells less than 5 (see Fennah 1956: 188, Fig. 54 a, d, g)	*Tambinia guamensis* Metcalf
–	Forewings with Cu_1_ forking basad of level of union of claval veins, with 14 apical cells, subapical cells more than 6 (see Fennah 1970: 77, Fig. 46)	*Tambinia pitho* Fennah
18	Body concolorous, without marks or different colors	19
–	Body suffused with marks or different colors	20
19	Vertex medially 1.4 times as long as maximum breadth, pronotum without short carinae between median carina and lateral margin (see Distant 1906: 277, Fig. 128)	*Tambinia inconspicua* Distant
–	Vertex medially 1.7 times as long as maximum breadth, pronotum with a pair of short carinae basally between median carina and lateral margins (see Fennah 1956: 189, Fig. 54 e, f, i)	*Tambinia sisyphus* Fennah
20	Forewings with nodal line suffused with pigmentation	21
–	Forewings with nodal line concolorous, without pigmentation	22
21	Vertex and pronotum with orange marks, nodal line suffused with fuscous (see Wilson 1986: 386, Figs 1, 3)	*Tambinia verticalis* Distant
–	Vertex, pronotum and mesonotum red, carinae green (see Wilson and Malenovský 2007, Fig. 3)	*Tambinia fasciculosa* Melichar
22	Forewings suffused with marks	23
–	Forewings without marks (see Melichar 1914: 86)	*Tambinia exoleta* Melichar
23	Vertex and pronotum finely marked with red spots (see Distant 1916: 48)	*Tambinia rubromaculata* Distant
–	Vertex marked with six red spots, pronotum with lateral carinae red (see Melichar 1914: 87)	*Tambinia venusta* Kirkaldy

### Tambinia conus sp. n.

urn:lsid:zoobank.org:act:B4955F97-4D85-485C-8200-345005DD0F1B

http://species-id.net/wiki/Tambinia_conus

Figs 1A 2A–H

#### Description.

 Body length (from apex of vertex to tip of forewings): ♂ 6.5 mm (N=1).

Colour. General colour tawny yellow, vertex (Figs 1A, 2A) with two short reddish stripes, pronotum (Figs 1A, 2A) with a pair of orange spots outside lateral carinae, median carinae on vertex and pronotum orange, mesonotum (Figs 1A, 2A) with a pair of orange spots beside lateral carinae near posterior margin, genae (Fig. 2B) with orange patch between eye and lateral margin of frons, forewings (Figs 1A, 2D) with many reddish spots marked from basal part to nodal line, tips of spines on hind tibiae and tarsi black.

Head and thorax. Head (Figs 1A, 2A, 2B) projecting before eyes approximately median length of eye, strongly dorsoventrally depressed. Vertex (Figs 1A, 2A) slightly longer in middle than the widest breadth (1.1: 1), distinctly longer than pronotum at midline (1.6: 1); anterior margin projected at an obtuse angle in dorsal view, lateral margins ridged and converged anteriorly; median carina thin and percurrent, with a pair of short sublateral carinae basally between median carina and lateral margins; posterior margin straight. Frons (Fig. 2C) longer in middle than the widest breadth (1.4: 1), disc flat and smooth, covered with sparsely microsetae (Fig. 2B); lateral margins sinuous, diverging from apex, slightly concave at level of eyes, then diverging further to reach their widest point before converging to the clypeus; median carina slender, gradually thinning and obsolete posteriorly, almost reaching to frontoclypeal suture. Clypeus (Fig. 2C) triangular, with broad median carina. Pronotum (Figs 1A, 2A) distinctly shorter than mesonotum in midline (0.4: 1), carinae strongly ridged, lateral carinae diverging posteriorly, median carina distinct, reaching posterior margin. Pronotum and mesonotum together medially 2.2 times as long as median length of vertex. Hind tibiae each with 2 distinct lateral spines; spinal formula of hind leg 5–5–2. Forewings (Figs 1A, 2D) relatively elongate and narrow, 2.7 times as long as maximum breadth, with corium smooth, not granulate, Sc+R forking at 2/5 apical, Cu_1_ forking after level of junction of claval veins, cell Sc with a short cross vein at its apical angle, with 13 apical cells and 6 subapical cells, claval veins uniting basad of middle of clavus.

Male genitalia. Pygofer (Figs 2F–H) narrow and relatively high, wider ventrally than dorsally, anterior margin moderately concave, posterior margin nearly straight on ventral half in lateral view. Anal tube (Figs 2F, 2G) distinctly elongate, surpassing to apex of gonostylus, ventral margin slightly bent ventrad in lateral view; lateral margins narrowing distad, apical margin distinctly forked in dorsal view; anal styles relatively short and stout, not surpassing apex of anal tube in dorsal view. Gonostylus (Figs 2F, 2H) very narrow, apical part dorsoposteriorly directed in lateral view; median conical process distinctly elongate and strong, sclerotized, nearly reaching to middle part of gonostylus in ventral view. Periandrium (Fig. 2F) distinctly short, ring-shape, with a long process directed caudad at ventral side, surround aedeagusat medially. Aedeagus (Fig. 2F) with shaft sinuate and apical half dorsoposteriorly directed in lateral view, apical part forking at endosoma, forming two process, which dorsal one distinctly longer than the ventral one; endosoma membranous, slightly expanded.

#### Material examined.

 Holotype ♂, PAPUA NEW GUINEA: Madang Province, Finisterre Range, Teptep, stream NE of town, 2100-2560 m, 23 Mar 1989, Stop #89-40A, D. H. Kavanaugh and G. E. Ball collectors, PAPUA NEW GUINEA EXPEDITION-1989 (CAS).

#### Etymology.

 This new species is named for the presence of a strong median conical process at apically inner margin of gonostylus (Figs 2F, 2H).

#### Distribution.

 Papua New Guinea.

#### Remarks.

 This species is similar to *Tambinia languida* Stål, 1859 collected from Sri Lanka, but can be distinguished from the latter in the vertex with two short reddish stripes, pronotum with a pair of orange spots outside lateral carinae, carinae of vertex and pronotum orange, mesonotum with a pair of orange spots beside lateral carinae near posterior margin, forewings with many reddish spots marked from basal part to nodal line and the frons with ratio of median length to the widest breadth 1.4:1 (in *Tambinia languida*, vertex and pronotum without pigmentation, mesonotum sometimes suffused with ochraceous, the frons with ratio of median length to the widest breadth 2:1, see Stål, 1859: 317; Melichar, 1914: 85).

### Tambinia macula sp. n.

urn:lsid:zoobank.org:act:AD6252E1-EF47-441F-A4CC-8F6D1A721CE8

http://species-id.net/wiki/Tambinia_macula

Figs 1B 3A–H

#### Description.

 Body length (from apex of vertex to tip of forewings): ♂ 5.6 mm (N=1).

Colour. General colour ocherous, vertex (Figs 1B, 3A) with median carina suffused reddish, the reddish extending from the sides, forming two reddish long stripes, its outer margins irregular, pronotum (Figs 1B, 3A) with a pair of reddish spots at disc depression between median and lateral carinae, frons (Fig. 3C) suffused with pale reddish, forewings (Figs 1B, 3D) with basal portion ocherous, with two red elongate marks near bases of sutural margins, many orange or red spots marked from basal part to nodal line, nodal line suffused with one transverse orange to red band, tips of spines on hind tibiae and tarsi black.

Head and thorax. Head (Figs 1B, 3A) projecting before eyes approximately median length of eye, strongly dorsoventrally depressed. Vertex (Figs 1B, 3A) about as long as broad, two times as long as median length of pronotum, anterior margin projected at an obtuse angle in dorsal view, lateral margins ridged and converged anteriorly; median carina thin and percurrent; posterior margin straight. Frons (Fig. 3C) longer in middle than the widest breadth (1.3: 1), disc slightly depressed, covered with sparsely microsetae (Figs 3B, 3C); lateral margins sinuous, diverging from apex, slightly concave at level of eyes, then diverging further to reach their widest point before converging to the clypeus; without median carina. Clypeus (Fig. 3C) triangular, without median carina. Pronotum (Figs 1B, 3A) distinctly shorter than mesonotum in midline (0.3: 1), carinae strongly ridged, lateral carinae diverging posteriorly, median carina distinct, reaching posterior margin. Pronotum and mesonotum together medially 2.1 times as long as median length of vertex. Hind tibiae each with 2 distinct lateral spines; spinal formula of hind leg 5–5–2. Forewings (Figs 1B, 3D) relatively broad, with basal portion semihyaline, thicker than apical portion, without granulation, 2.7 times as long as maximum breadth, Sc+R forking about medially, Cu_1_ forking after level of junction of claval veins, cell Sc with a short cross vein at its apical angle, with 12 apical cells and 5 subapical cells, claval veins uniting distad of middle of clavus.

Male genitalia. Pygofer (Figs 3E, 3G, 3H) narrow and high, wider ventrally than dorsally, anterior margin concave medially, posterior margin produced caudad in lateral view. Anal tube (Figs 3E, 3F) relatively elongate, ventral margin nearly straight and directed caudad in lateral view; lateral margins slightly diverging distad, apical margin concave in dorsal view; anal styles relatively long, distinctly surpassing apex of anal tube in dorsal view. Gonostylus (Figs 3E, 3G, 3H) elongate, basal half broad and apical half abruptly narrow in lateroventral view; median conical process very thin and slender, sclerotized in ventral view. Periandrium (Fig. 3E) tube-like, distinctly sclerotized, with a short process directed ventrad at dorsal apex, surrounding aedeagus medially. Aedeagus (Fig. 3E) with shaft very long and thin, simple tubule, sinuate and its apex directed caudad in lateral view, endosoma indistinct.

#### Material examined.

 Holotype ♂, MALAYSIA: Banaakan Borneo, 1927.I, Pemberton Coll. (CAS).

#### Etymology.

 This new species is named for the presence of many reddish markings on vertex, pronotum and tegmina (Fig. 1B).

#### Distribution.

 Malaysia (Borneo).

#### Remarks.

 This species is similar to *Tambinia atrosignata* Distant, 1906, but can be distinguished from the latter in vertex with two reddish long stripes, pronotum with a pair of reddish spots, forewings with basal portion ocherous, with two red elongate marks near bases of sutural margins, many orange or red spots marked from basal part to nodal line and nodal line suffused with one transverse orange to red band.

### Tambinia robustocarina sp. n.

urn:lsid:zoobank.org:act:4583165F-DBE6-4F7A-AA36-FC1FBF6369FF

http://species-id.net/wiki/Tambinia_robustocarina

Figs 1D 4A–G

#### Description.

 Body length (from apex of vertex to tip of forewings): ♂ 6.8 mm (N=1).

Colour. General colour tawny yellow, forewings (Figs 1D, 4D) with two fuscous elongate marks near bases of sutural margins, nodal line suffused with pale brown marks, many fuscous spots marked from nodal line to apex, tips of spines on hind tibiae and tarsi black.

Head and thorax. Head (Figs 1D, 4A) projecting before eyes approximately 3/5 median length of eye, not strongly dorsoventrally depressed. Vertex (Figs 1D, 4A, 4B) distinctly shorter in middle than the widest breadth (0.6: 1), distinctly longer than pronotum at midline (1.7: 1), anterior margin convex, broadly callused, uniting with base of frons to form smooth surface, lateral margins ridged and converged anteriorly, median carina long and percurrent, thickened and broad, posterior margin straight. Frons (Fig. 4C) slightly longer medially than greatest width (1.3: 1), disc flat and smooth, covered with sparsely microsetae (Fig. 4B), lateral margins diverging to below level of eyes, distinctly callused; median carina with basal part strongly broad and thickened, not reaching to frontoclypeal suture, obsolete on level of antennae. Clypeus (Fig. 4C) triangular, with distinctly broad median carina. Pronotum (Figs 1D, 4A) distinctly shorter than mesonotum in midline (0.2: 1), carinae broadly ridged, lateral carinae diverging posteriorly, median carina distinctly thickened and broad, reaching posterior margin. Pronotum and mesonotum together medially 3.0 times as long as median length of vertex. Hind tibiae each with 2 distinct lateral spines; spinal formula of hind leg 5–5–2. Forewings (Figs 1D, 4D) relatively elongate and narrow, 3.0 times as long as maximum breadth, with corium smooth, not granulate, Sc+R forking at apical 2/5, Cu_1_ forking after level of junction of claval veins, with 11 apical cells and 6 subapical cells, claval veins uniting at about middle of clavus.

Male genitalia. Pygofer (Figs 4E–G) irregular subquadrate in lateral view, anterior margin concave on dorsal 1/3, posterior margin produced caudad in lateral view. Anal tube (Figs 4E, 4F) relatively elongate, ventral margin slightly bent ventrad in lateral view; lateral margins convex medially then narrowing distad, apical margin slightly concave in dorsal view; anal styles relatively long and narrow, surpassing apex of anal tube in dorsal view. Gonostylus (Figs 4E, 4G) elongate, but not surpassing to apex of gonostylus, apical half narrow and basal half broad in lateral view; median conical process very small, sclerotized in ventral view. Periandrium (Fig. 4E) distinctly elongate and slender, tube-like, distinctly sclerotized, with a short process directed caudad at dorsal apex, surrounding aedeagus subapically. Aedeagus (Figs 4E, 4F) with shaft thin and tubular, arched and its apex directed ventrad in lateral view, endosoma membranous, moderately expanded, with two, anteroventrally directed, spinous processes on right side in lateral view.

#### Material examined.

 Holotype ♂, MALAYSIA: Malaysia: Sabah: 25 km N Tambunan, 1500 m, 1983.IX.3, at black light, G. F. Hevel & W. E. Steiner (USNM).

#### Etymology.

 This new species is named for the presence of a robust median carina on the vertex (Figs 1D, 4A).

#### Distribution.

 Malaysia (Sabah).

#### Remarks.

 Based on the following combination of characters: head relatively short, not strongly dorsoventrally depressed, broadly produced anteriorly; vertex with median carina strongly thickened and broad; pronotum with median carina relatively broad and frons with basal part of median carina strongly broad and thickened, this species and the four previously described species, *Tambinia menglunensis*, *Tambinia rubrolineata*, *Tambinia similis* and *Tambinia theivora* form a very distinct group within *Tambinia*.

In external appearance, this species is similar to *Tambinia similis* (Fig. 1C) and but differs from the latter in the median carina on vertex long and percurrent, thickened and broad, but not spatula-like, forewings relatively broad, nodal line relatively near middle and cell Sc without a short cross vein at its apical angle. This species is also similar to *Tambinia menglunensis* (see Men and Qin, 2009: 263, Figs 1, 2), but differs from the latter in the obsolete spots and markings on the vertex, pronotum, mesomotum and forewings, median carinae on vertex, pronotum and frons strongly thickened and broad, and gonostylus with median conical process very small.

### Tambinia sexmaculata sp. n.

urn:lsid:zoobank.org:act:56274E10-6B5F-41CC-9DB7-563446EC4CD2

http://species-id.net/wiki/Tambinia_sexmaculata

Figs 1E 5A–H

#### Description.

 Body length (from apex of vertex to tip of forewings): ♂ 6.2 mm (N=1), ♀, 6.6–6.8 mm (N=2).

Colour. General colour tawny yellow, vertex (Figs 1E, 5A) with six red spots, genae (Fig. 5B) with orange patch between eye and lateral margin of frons, forewings (Figs 1E, 5D) with two pairs of red spots near bases of sutural margins and distad of level of union of claval veins, relatively, tips of spines on hind tibiae and tarsi black.

Head and thorax. Head (Figs 1E, 5A) projecting before eyes 1.2 times as long as median length of eye, strongly dorsoventrally depressed. Vertex (Figs 1E, 5A) distinctly longer in middle than the widest breadth (1.1: 1), distinctly longer than pronotum at midline (2.3: 1); anterior margin projected at an obtuse angle in dorsal view, lateral margins ridged and converged anteriorly; median carina thin and percurrent, with a pair of short sublateral carinae basally between median carina and lateral margins; posterior margin nearly straight. Frons (Fig. 5C) longer in middle than the widest breadth (1.6: 1), disc flat and smooth, covered with very sparsely microsetae (Figs 5B, 5C); lateral margins sinuous, diverging from apex, slightly concave at level of eyes, then slightly diverging to reach their widest point before converging to the clypeus; median carina long and slender, nearly reaching to frontoclypeal suture. Clypeus (Fig. 5C) triangular, with distinct median carina. Pronotum (Figs 1E, 5A) distinctly shorter than mesonotum in midline (0.3: 1), carinae strongly ridged, lateral carinae moderately diverging posteriorly, median carina distinct, reaching posterior margin. Pronotum and mesonotum together medially 2.0 times as long as median length of vertex. Hind titiae each with 2 distinct lateral spines; spinal formula of hind leg 4–5–2. Forewings (Figs 1E, 5D) relatively elongate and narrow, 2.8 times as long as maximum breadth, with corium smooth, not granulate, Sc+R forking at 2/5 apical, Cu_1_ forking at level of junction of claval veins, with 12–13 apical cells and 5 subapical cells, claval veins uniting distad of middle of clavus.

Male genitalia. Pygofer (Figs 5F–H) moderately broad, anterior margin concave on dorsal 1/3, posterior margin convex caudad in lateral view. Anal tube (Figs 5F, 5G) distinctly elongate, almost surpassing to apex of gonostylus, ventral margin slightly curve dorsad in lateral view; lateral margins concave medially then diverging from apex, apical margin distinctly concaved in dorsal view; anal styles long and strong, surpassing apex of anal tube in dorsal view. Gonostylus (Figs 5F, 5H) very narrow, expanded subapically then narrowing to apex, directed caudad in lateral view; median conical process distinct, relatively short. Periandrium (Figs 5F, 5G) distinctly elongate and sclerotized, tube-like, surrounding aedeagus medially, with a long, sinuate process at left side, dorsoposteriorly directed. Aedeagus (Figs 5F, 5G) with shaft tubular, apical part abruptly curved through approximately 30˚, directed to right; endosoma indistinct.

#### Material examined.

 Holotype ♂, AUSTRALIA: Kuranda N. Q. Australia, 1904.VIII.10. Koebele, W. M. Giffard Collection (CAS). Paratypes. 2♀♀, the same data with Holotype (CAS).

#### Etymology.

 This new species is named for the presence of six reddish markings on vertex (Figs 1E, 5A).

#### Distribution.

 Australia (Kuranda).

#### Remarks.

 This species is similar to *Tambinia conus* but can be distinguished from the latter in the vertex with six red spots, forewings with two pairs of red spots and by the male genitalia structure (Figs 5F–H), especially the shape of anal tube, median conical process of gonostylus relatively small, periandrium relatively long, with a long, sinuate process at left side, dorsoposteriorly directed, and the shaft of aedeagus apical part abruptly curved through approximately 30˚, directed to right.

## Supplementary Material

XML Treatment for Tambinia

XML Treatment for Tambinia conus

XML Treatment for Tambinia macula

XML Treatment for Tambinia robustocarina

XML Treatment for Tambinia sexmaculata
